# Predictors of disability in children with inflammatory arthritis, two and three years after first presentation to paediatric rheumatology. Results from the childhood arthritis prospective study (CAPS)

**DOI:** 10.1186/1546-0096-9-S1-O42

**Published:** 2011-09-14

**Authors:** R Carrasco, J Cobb, E Baildam, H Foster, J Gardner-Medwin, A Chieng, L Wedderburn, J Davidson, K Hyrich, W Thomson

**Affiliations:** 1 Arthirtis Research UK Epidemiology Unit, The University of Manchester, Manchester, UK; 2 Royal Liverpool Children's Hospitals NHS Trust, Liverpool, UK; 3 Newcastle Hospitals NHS Trust, Newcastle; 4 Royal Hospital for Sick Children, Glasgow; 5 Central Manchester University Hospital NHS Trust, Manchester; 6 Institute of Child Health, UCL, London, UK

## Background

Inflammatory arthritis (IA) in children is a chronic and often disabling disease with variable outcome. Up to 1/3 of children are reported to have active disease progressing into adulthood. The Childhood Arthritis Prospective Study (CAPS) is a prospective longitudinal inception cohort study which aim is to identify genetic and environmental predictors of short- and long-term outcome of childhood IA. Previously, we reported that the strongest predictor of persistent disability at one-year (as measured by the CHAQ) was moderate to severe disability at first presentation. The objective of this study was to extend the one year analysis to look at both two and three year outcomes.

## Methods

CAPS recruits children <16 years with new inflammatory arthritis persisting for ≥ 2 weeks from five referral centres. Demographics, disease features, active and limited joint counts, CHAQ, physician’s global assessment (PGA), parent’s general evaluation of well-being (PGE), erythrocyte sedimentation rate (ESR) and treatment, are collected at first presentation to a paediatric rheumatologist, 6 months, then yearly. Independent predictors of moderate to severe CHAQ (CHAQ≥0.75) at 2 and 3 years in children with JIA were identified using multivariable logistic regression models. Imputation was performed in order to account for missing data.

## Results

To date, 1059 children have been recruited; 901 had reached one year, 852 two years and 618 three years of follow-up. Median age at presentation was 7.7 years, 63% girls. ESR, Joint counts, PGE and PGA score decreased every year for the majority of patients. Median CHAQ score was 0.75 (IQR: 0.125-1.375) at presentation and decreased to 0.25 (IQR: 0.0-1.0) at 1 year, 0.125 (IQR: 0.0-0.875) at 2 years and 0.125 (IQR: 0.0-0.75) at 3 years. Fifty percent of the children had CHAQ score ≥0.75 at presentation, 33% at 1 year, 32% at 2 years and 28% at 3 years. CHAQ≥0.75 at presentation was the strongest predictor of moderate to severe disability at one (OR 2.83 95% CI: 1.48, 5.37), two (OR 3.49 95% CI: 1.73, 7.08) and three years (OR 6.47 95% CI: 2.35, 17.8). After missing data imputation an additional predictor, PGE, was found.

## Conclusions

Majority of children presenting with IA show continued improvement in the three years following first presentation, with many having no active or limited joints. There are still a significant proportion (28%) with moderate to severe disability. CHAQ score ≥0.75 at presentation is the strongest predictor of outcome at two and three years although an additional predictor, PGE may also be important. Future analysis will look at predictors of outcome, taking into outcome treatment.

